# On Diseases of the Liver

**Published:** 1846-01

**Authors:** 


### Art. III.

*On Diseases of the Liver.* By George Budd, m.d. f.r.s. Professor of Medicine in King's College, London, and Fellow of Caius' College, Cambridge.—*London,* 1845. 8vo. pp. 402.

Dr. Budd commences his volume with observing that "there are no other diseases of such frequent occurrence, which it is so difficult to discriminate, and for the treatment of which, the medical practitioner has so few trustworthy guides," as diseases of the liver. (p. 1.) Surely diseases of the cerebro-spinal system must be excepted from this remark, of which the diagnosis is as difficult, the pathology as obscure, as any of those of the liver, and which, in a therapeutical point of view, are even more intractable and more perplexing. Still we admit that there is considerable truth in Dr. Budd's observation, and no one of any practical experience can fail to be sensible of the frequent unsatisfactory results of even the most carefully-considered treatment of hepatic diseases.

Anatomy of the liver. We presume our readers to be acquainted with all the important points connected with the anatomy and physiology of the liver. Of these the author gives a sufficiently distinct sketch, for which we must refer to the work itself. We may here observe that a few points of curiosity, rather than of practical interest or importance, are still unsettled, in regard to the structure of the liver. Thus it is still *sub lite,* whether the blood of the hepatic artery passes into the extreme branches of the portal vein, *before* entering the hepatic veins. Mr. Kiernan believes that it does. It is not obvious that any change in therapeutics would be rendered necessary by the absolute decision of the question one way or another. Müller, among others, supposed that what has been so long called the capsule of Glisson, was prolonged, as a sheath of cellular membrane, investing each lobule and separating it from contiguous lobules. Mr. Bowman, whose opinion is paramount with Dr. Budd, thinks he has discovered that there is no areolar tissue between the lobules. These lobules consist of masses of nucleated cells, which, as well as the ducts (which it seems also to secrete,) are the elaboratories of the bile. Among doubtful points, is the exact mode of connexion of the hepatic ducts with these nucleated cells.

As the nucleated cells, along with the capillaries in the meshes of which the cells lie, compose the lobules, and as the lobules compose the substance of the liver, with the exception of the several systems of vessels, it follows that nucleated cells and capillary vessels compose nearly the whole of the organ.

Mr. Kiernan is of opinion that no arteries enter the lobules.

In the nucleated cells Mr. Bowman thinks he has discovered globules of oil or fat, and believes that it is in the increase of the size and number of these globules, that the fatty degeneration of the liver of phthisical and other patients consists. It is probable that these lobules must be, in some way, disintegrated or dissolved before they pass from the nucleated cells into the hepatic ducts.

The Bile.—*Composition and properties.* With the appearance and properties of the bile, we must presume our readers to be acquainted. The degree of fluidity and its colour are extremely various, so that it is, perhaps, impossible to determine exactly what should be considered normal as regards either of these qualities. The darkness of tinge and the degree of dilution of bile depend on the length of time it has remained in the hepatic ducts, or in the gall-bladder. Consequently, several hours after the *completion* of digestion, and more especially after protracted abstinence from food, it is peculiarly dark and inspissated. At other times, its colour varies from yellow to a deep brown-black ; but there is generally a shade of green mixed with the yellow, more especially in the bile of the gall-bladder. It feels oily and slightly adhesive, tastes bitter, but has little or no smell. It is readily raised, by agitation, into froth or foam. Schültz stated the specific gravity of ox bile at 1026 to 1030 : and we are not aware that there is any authentic determination as to the gravity of human bile. The same physiologist had stated bile to be alkaline ; and that one ounce of it, when tolerably inspissated, required one drachm of acetic acid for its neutralization ; but Bouisson and Dr. Kemp have more recently determined that when normal and fresh, bile is neutral. When concentrated, it contains grayish-white or greenish particles, and some of the prism-like cells which bestud the mucous membrane of the gall-bladder. To these may be added, cholesterine scales and globules of oil. The former, Dr. Budd seems to think, seldom or never appear except as a consequence of disease of the gall-bladder. The following, according to Berzelius, is the constitution of cystic ox bile :

| | |
|---|---|
| Water | 90·44 |
| Biliary matter with fat | 8·00 |
| Mucus | 30 |
| Osmazome, chloride of sodium, lactate of soda | 74 |
| Soda | 41 |
| Phosphate of soda, of lime, and traces of a substance insoluble in alcohol | 11 |
| | 100·00 |

Cholesterine, dissolved and in small quantity, appears also to exist in bile, but does not, unless in cystic disease, appear in the form of scales.

*Sources and uses of the bile.* The views entertained of the sources and uses of the bile have, of late years, undergone very important alterations. At one time, this fluid was regarded as almost wholly excrementitious; another supposed principal use was to promote the peristaltic action of the intestines. The present belief is, that the bile, far from being to a great extent excrementitious, is almost entirely *re*-crementitious ; and though it seems probable that the evacuatory movement of the intestines is in part owing to this "natural purgative," as Dr. Budd terms it (p. 30,) yet doubtless that effect is less due to any isolated action of the bile, than to the normal stimulus of the fecal mass, as a whole.

The bile, in common with the excretions of the lungs and kidney, is probably, to a great extent, derived from the decomposition of the tissues of the body, but part of it also is, no doubt, educed from non-nitrogenized articles of food. Carbon is the substance chiefly eliminated by the liver, in like manner as it, along with hydrogen, is the chief constituent of the pulmonary excretion. But an important distinction obtains between the two cases. While the carbon of the lungs is united to oxygen, and excreted in a non-combustible state, the carbon of the liver is non-oxygenized, is still combustible, and is intended, not for excretion, but absorption. But the fact that the two organs secrete carbon, gives them a complementary relation, of which we avail ourselves in therapeutics.

The quantity of bile secreted by a man, daily, has been variously estimated, from a very few ounces to 24 ounces. Before the important and large recrementitious uses of the fluid were ascertained, and when the comparatively small quantity evacuated in the fæces was believed to form the whole amount, the bile secreted in the twenty-four hours was computed at from 3 to 5 or 7 ounces. Yet even Haller, who guessed that the bile served other purdoses than excrementition, estimated the quantity at from 17 to 24 ounces; and this calculation Schültz and Liebig adopt. There appear to us reasons (which we shall not here detail) for believing that from 11 to 14 ounces is the average quantity of the healthy adult secretion of bile, in the space of four-and-twenty hours.

To return now to the consideration of the uses of the bile. These seem to be various. First, then, the liver is, *in part,* an excrementory organ. The resin and the colouring matter are excrementitious matters. Secondly, the liver is a depuratory organ. The abdominal circulation returns through it, and, as Dr. Budd justly observes, (p. 28,) "the blood which has come from the stomach and intestines must necessarily be charged with many impurities besides those derived from the mere decay of the tissues. Along the extensive mucous tract with which everything we eat and drink is brought in contact, absorption is constantly going on, and various matters must therefore enter the portal vessels, not fit by their nature to form blood, or to serve any other purpose in the body. Many of these substances are removed from the blood in its passage through the liver." The part which the bile plays in digestion is more obscure and uncertain. Dr. Budd agrees with those who think that the share which the liver has in assimilation, has been over-rated. According to the most recent and approved views of physiologists and chemists, the *solution* of our food is all that is required, and not, as was formerly supposed, a mysterious *production* from it of albumen, fibrin, and casein, which it did not previously contain. For we know that food, both animal and vegetable, *does* contain, ready formed, these constituents of the blood. Now, this solution is effected, that is, chymification is completed, without the aid of the bile. It was till lately supposed that one use of the bile was to neutralize the acid of the chyme, and this notion rested on the assumed fact of the bile being alkaline. But if Bouisson's and Dr. Kemp's late researches are accurate, the bile is neutral, and therefore the use referred to is imaginary; unless, as Dr. Budd (p. 30) conjectures, the bile is decomposed in its passage along the bowels, its soda uniting with the acid of the chyme. Dr. Prout's opinion (see Stomach and Renal Diseases, p. 467, ed. 1843,) seems to be, that the chloric acid of the chyme is derived from the common salt existing in the blood and in the stomach: that the salt suffers decomposition; chloric acid is set free, and the soda remains in the stomach, or is absorbed (both of these probably happen) again into the blood. Part of it, a small portion, goes, as Dr. Prout thinks, to maintain the weak alkalinity of the blood: the greater portion returns to the liver, there to be eliminated and to reunite, in the duodenum, with chloric acid. Thus, as it were, an endless cycle of union and separation of part at least of the constituents of the common salt of the blood, takes place. And may we not suppose that something of the same kind happens as regards the carbon of the decomposed tissues, elicited in the bile? Having undergone some purifying process in the course of that elimination, it is fitted for again serving as *respiratory* food, and is, if we may use the phrase, economically reabsorbed. This, supposing the present theories of respiration and animal heat to be true, appears to us the true explanation and history of the series of changes going on in the duodenum.

It must not, however, be supposed that the bile furnishes anything approaching to the principal proportion of the carbon, excreted by the lungs and skin. The quantity of carbon supposed to be eliminated by these two channels, is said to average $13\frac{9}{10}$ ounces daily, (this is Liebig's computation.) Now, the carbon of the bile, even at the highest estimate, does not exceed three ounces; and if our calculation of from 11 to 14 ounces be regarded as expressing the average daily secretion of bile, the carbon contained in it is about one ounce only.

*Agents affecting the bile.* At the conclusion of his introductory chapter, and before he proceeds to the main business of the work, Dr. Budd makes some pathological and therapeutical observations which perhaps might have been reserved for a subsequent part. Among cholagogue medicines he specially mentions mercury, iodine, muriate of ammonia, and taraxacum. For our parts, we have never observed peculiar cholagogue properties in muriate of ammonia, nor yet in rhubarb, which Dr. Budd subsequently instances. Bonnet enumerates rhubarb as one of the irritating substances, and unskilfully employed by some practitioners in hepatic disease. We cannot say we have found it to be irritating; assuredly we have not found it to be specially cholagogue. We are surprised that in the brief list of drugs particularly adapted for "rendering the liver more active, and increasing in this way the secretion of bile," (p. 35,) Dr. Budd should have omitted colchicum, and even colocynth, but the former more particularly. Perhaps next to mercurial preparations, there is no agent more remarkable, in that way, than the one just named. Nitric acid also, not here referred to by Dr. Budd, seems in some cases notably to promote the action of the liver.

Diseases of the liver. The first morbid condition which Dr. Budd treats of is congestion. (p. 38.)

*Congestion.* The common and usual form of congestion of the liver is that of the hepatic vein and its capillaries; and that form may be caused by any disease in the heart or lungs tending to obstruct the return of blood by the hepatic veins. If, while the hepatic veins and their capillaries are thus congested, the portal vein and the capillaries immediately branching from it are empty, the appearance called mottled liver is presented, caused by the central vessels of the lobules being full of blood, while those on their margins are void of it. And just in proportion as the vessels continue to be distended in a direction distal from the heart, and as the portal capillaries which form the margin of the lobules become filled gradually, will the mottled appearance merge into a homogeneous redness. And now, in addition to sanguineous, will biliary congestion begin to take place; and this plainly in consequence, in the majority of cases at least, of the pressure of the distended blood-vessels on the minute branches of the biliary ducts, whereby the discharge of bile along these ducts is impeded. The general result of this state of things is hyperemia, and a state of distension of the biliary vessels analogous to hyperemia, but for which we have no exact name. The liver, of course, becomes enlarged, and its colour, when an incision is made, is a deep reddish-brown or black.

The diagnostic indications of this state are, the dropping of the liver several inches (more or less, according to the degree of enlargement,) below the ribs, and a fulness felt by the patient and perceived by the physician in the right hypochondrium. There is not "in general" pain (p. 41); never, we should say, in this simple form of congestion, until its prolongation begins to give rise to inflammatory action. And even then pain is hardly or not at all experienced, until the peritoneal investments begin to suffer, and the affection becomes what Bonnet calls hepato-peritoneal. (See Article V. of our Number for July, 1843.)

Besides affections of the heart and lungs, the hot stage of ague, and occasionally purpura hæmorrhagica, (if we are to credit Dr. Budd,) are attended with hepatic congestion. Andral states (p. 10, tome 4, of his Clin. Med. ed. 1827,) that he has often observed great congestion of the liver in persons dead of scorbutic disease. In these cases the spleen was also hyperemic, but the congestion of neither organ had any connexion with inflammatory action. He seems to be of opinion that it is in diseases attended with a great diminution of the fibrin of the blood, that congestion of the liver and spleen are most apt to occur.

Congestion confined to the portal system is, according to Mr. Kiernan, who alone gives any account of it, a rare affection, occurring, so far as his experience goes, only in children.

Dr. Budd's remarks on the treatment of congestion of the liver, are extremely concise, and as vague and general as possible. (p. 43.) In congestion depending on obstructed circulation through the heart, he advises bleeding, purgatives, diuretics, rest,—those measures, in short, which peculiarly relieve cardiac disease. He does not mention blistering and friction over the heart, yet these are important means. "When the liver," he remarks, "cannot free the blood from the principles of bile, or the skin becomes sallow, the patient should carefully abstain from rich meats and fermented drinks, which would render the liver still more inadequate to its office, and increase the biliary disorder." (p. 43.)

Purpura hæmorrhagica, scorbutic disease, and any affections tending to diminish the fibrin of the blood, require, we need not remark, the mineral acids and the bitter extracts. We have witnessed great advantage from the compound infusion and spirit of armoracia, taken twice or thrice a day, with ten drops of nitric or chloric acid added to each dose.

We may remark, before quitting this lesion, that in long-continued congestion of the liver, the nucleated cells, the seats of the biliary secretion, are apt to be obliterated, or at least to suffer in their structure, and, in this way, the function of the liver is liable to be materially and perhaps permanently impaired.

Dr. Budd devotes chapter second to the consideration of "Inflammatory Diseases of the Liver." The first section of the chapter is allotted to *suppurative* inflammation and abscess. Before proceeding, however, to the discussion of the matters just named, he touches on and condemns the usual classification of inflammatory diseases of the liver. The wonted division of such diseases is into acute and chronic, which he characterizes as "essentially faulty," (p. 46;) and on the very just grounds, that it is inaccurate, and corresponds not with, or rather misleads us in regard to, the actual facts. Deep-seated inflammation of the liver may, if not very extensive, run rapidly into abscess, *without* very striking symptoms of any kind, and might therefore be called "*chronic* during the life of the patient, while inflammation involving the surface of the liver, even of such a kind as causes the slow effusion of coagulable lymph only, will be attended with well-marked local symptoms, with great pain and tenderness, and would be termed *acute*." (p. 46.)

The author therefore thinks that, in the case of liver-disease, as well as of every other, morbid affections should as nearly as possible be named from their causes. He therefore divides inflammatory affections of the liver into—1, Suppurative; 2, Gangrenous; 3, Adhesive.

*Suppurative inflammation.* Perhaps this section on suppurative inflammation and abscess of the liver is the most interesting and important in the entire volume before us. Dr. Budd's object is to show, that by far the majority of cases of abscess of this viscus are owing to "suppurative inflammation of some vein, and the consequent contamination of the blood by pus." (p. 49.) It had been long observed that, subsequently to large wounds or surgical operations, and where suppuration had taken place, pus often formed in remote parts, as, for example, in the interior of joints, in the lungs, in the liver. This was called and supposed to be a *deposit* of pus: it was imagined that the pus was not formed at the place where it was found, but was brought thither from the primary suppurating surface by the vessels, and then deposited or excreted. But when it was ascertained that pus-globules are nearly twice as large as blood-globules, it became manifest that if the vessels deposited the former, they must, *à fortiori*, let the latter also escape. But in these secondary abscesses, while there was purulent, there was no sanguineous extravasation or deposition. It hence became manifest that the pus must be *formed* at the place where it was found, by the usual process of a local inflammation. The experiments of MM. Dance and Cruveilhier and of Dr. Saunders also showed that the veins must be the channel which transport the globule or globules of pus, which, entering these at some suppurating surface, and becoming arrested in the minute capillary vessels of the lungs or liver, cause those secondary abscesses of which these two organs, both from their peculiar structure and from the fact that all the blood of the body passes through them, are so frequently and remarkably the seat. Now, for the important practical elucidation derivable from the explanation just given, in regard to hepatic abscess so often consequent on dysenteric affections, and on wounds or diseases of the intestines, in which suppuration had taken place.

The author gives a good many cases from Cruveilhier, Andral, Louis, Annesley, and from his own practice on board the Dreadnought, illustrative of the relation of cause and effect, in the majority of cases, between ulcerated states of the intestinal mucous membrane and hepatic abscess. And, in our opinion, he most satisfactorily establishes this connexion. Louis, Andral, and Annesley notice the connexion, and, indeed, it had been long observed; but none of them appear to have surmised the nature of it. Dr. Budd would appear to hold (p. 62) that even in cases where hepatic disease seems to *precede* the dysentery and to cause it, yet that the formation of the hepatic abscess is still consequent on, and subsequent to, the dysenteric affection. In India, there are often cases which commence with biliary disturbance; by and by, fatal and rapid dysentery ensues, and the patient, on post-mortem inspection, is found with abscess of the liver, fully formed. Now, it might at first be suspected that the formation of the abscess, or the peculiar form of inflammation which caused it, preceded the dysentery.* If we understand Dr. Budd aright, (pp. 61-3,) the contrary is the case. The primary hepatic affection was *not* suppurative, but *adhesive* inflammation, the result of spirit-drinking, and liable to terminate in induration and cirrhosis,—seldom or never in *abscess*. But, possibly, the passage of acrid bile, the result of this primary affection, may have caused or predisposed to dysenteric inflammation, and during the resulting suppuration of the intestine, a globule (for often one seems sufficient) or globules, passing up a hemorrhoidal or other intestinal vein, has become arrested in the portal capillaries of the liver, inducing suppurative inflammation with abscess, in addition to the prior-existing adhesive inflammation of the organ.

But it is not necessarily dysenteric affections of the bowels alone, and the consequent ulceration; but ulceration from any cause, and situated in any part, *distal* of the liver, in consequence of which the veins proceeding from the part discharge into the *portal vein*, which may give rise to hepatic abscess. Accordingly we have (at pp. 65-7) cases referred to by Dr. Budd, in which the ulceration was seated in the stomach, in the gall-bladder, and hepatic ducts respectively. The author cites a case of Mr. Busk's, in which the splenic vein was the seat of the primary suppuration or ulceration.

* This view was taken many years ago by Dr. Archibald Robertson in his excellent Thesis (Edin. 1817,) " De Dysenteria regionum Calidarum."

At pp. 70-1-2-3, the author proceeds to confirm the views which we have now described, but there is nothing in this part requiring particular remark.

The "changes of structure," to which suppurative inflammation of the liver leads, is next considered, (pp. 74 et seq.)

Suppurative inflammation of the liver commences in, and often does not extend beyond, the lobular substance. If, indeed, the inflamed part approach, in any direction, the peritoneal surface of the organ, or be closely contiguous to a trunk of the hepatic vein, adhesive inflammation of the peritoneal lining, in the former case, and internal inflammation of the hepatic venous branch, in the latter case, may ensue. However, these secondary results are not usual. Dr. Budd has never seen any branch of the portal vein thus secondarily involved, though Dr. Russell of Birmingham has. The author attributes this difference to the circumstance of the coats of the branches of the portal vein being thicker than those of the hepatic, and also surrounded by areolar tissue.

The abscess or abscesses, if they have led to a *speedily* fatal issue, are bounded simply by red and softened parenchyma; and it may be laid down as a general rule, that, in proportion to the length of time the abscess has existed, (supposing the consequence not to be immediately fatal,) do the walls of the abscess assume more and more density and thickness. Sometimes, however, the contents of the cyst augment, and the pressure causes irritation of the internal walls, followed by ulceration and ultimate gradual extension of the abscess. The abscess, may of course, discharge in any direction ; as it reaches the surface of the liver, it excites inflammation in the hepatic peritoneum, by which adhesion between this and the parietal or diaphragmatic peritoneum is effected : or else the adhesion takes place between the hepatic surface and that of the stomach, duodenum, or bowels ; or the abscess may discharge, no such adhesions having been formed, directly into the abdominal cavity. This termination, as it is more hazardous, so it is rarer than the others.

The sizes of these abscesses are sometimes amazing. In one of Annesley's cases, the abscess contained 90 ounces : in one of Dr. Inman's of Liverpool, the quantity was 13 pints!

The *symptoms* of suppuration of the liver and of the formation of abscess, are generally obscure or rather equivocal, and sometimes extremely so. Though there occur occasionally, as concomitants of inflammation tending to abscess, pain, fulness in the hepatic region, and jaundice, yet all of these may be absent, or present in an almost unappreciable degree only, and may moreover be caused by other affections ; such as irritation or inflammation of the gall-ducts. When the abscess or abscesses are small and central, and when they do not cause any general obstruction to the passage of the bile, neither tumefaction nor jaundice ensue ; nor, in the same circumstances, is pain usually felt. Our diagnosis will, however, be materially facilitated should dysentery be present, or there be any suppurating mucous or cutaneous surface, or any extensive wound, accidental or surgical. Pain in the hepatic region, or fulness there, or jaundice occurring in these circumstances, must awake our anxiety and suspicion. The occurrence of a well-marked rigour would, of course, give additional force to other proofs or presumptions.

As regards pain in the right shoulder, which Dr. Budd considers in connexion with hepatic abscess, it occurs, according to our experience, less in acute than in chronic affections, and in all forms of hepatic disease or irritation. Andral and Louis appear to doubt whether it is ever present except when the lung or pleura is affected, and to place little or no faith in it as a sign of *hepatic* disease. It is usually said that it can indicate inflammation of the *convex* surface and of the right lobe of the liver alone, but it was present in a case of Andral's, cited by Dr. Budd himself, in which the abscess was on the *under* surface of the right lobe. "The pain," observes the author, "is, in fact, as it has always been represented to be, a *sympathetic* pain, like the pain of the knee from disease of the hip." (p. 84.) Dr. Budd is, no doubt, aware, that the pain is *accounted* for from the fact, that the phrenic nerve is derived from the fourth cervical nerve, and that this nerve also supplies the muscles and integuments of the neck and shoulder: it follows that, if the diaphragm should be irritated or stretched by enlargement of, or inflammatory action in, the liver, the nerve named from it may be affected; and, circuitously, the branches of the fourth cervical nerve which go to the neck and shoulder. We have often met with cases of hepatic disease in which the pain was felt in the left shoulder, and about the scapular muscles: it may be from the centre or left portion of the diaphragm having been peculiarly affected.

As to rigidity of the right rectus muscle, stated by Mr. Twining to be one of the most certain indications of *deep-seated* (?) abscess of the liver, we, for our parts, have never met with it, unless when the *parietal peritoneum participated in the inflammation of the subjacent hepatic peritoneum.* Then there was a slight apparent tension, probably owing to irritation of the rectus muscle. But why deep-seated abscess of the liver should cause rigidity of this muscle, unless that abscess, by enlarging greatly the bulk of the organ, gave rise to distension of the muscle, and thus elicited natural or spasmodic contraction of it, it were difficult to divine.

As to cough and vomiting, both of which Louis distrusts as symptoms of hepatic abscess, and which Dr. Budd confides in, describing them merely as "sympathetic disorders, depending solely on irritation of the liver," (p. 85,) the former is to be accounted for in the same way as the pain of the shoulder, the cough being simply caused by spasmodic action of the diaphragm, in consequence of irritation of its nerve by the adjacent enlarged or inflamed viscus; the latter occurs when the irritation of the liver has involved the duodenum, and when, besides, this intestine is excited by the acrid bile, which, when inflamed, the liver is apt, in the first stage of inflammatory action, plentifully to excrete.

At page 89, occurs a remark so extraordinary that we must quote, and we shall do so without a single observation. "The fatalness [of hepatic abscess] has no relation to the outlet by which the matter is discharged. I have met with several cases in which the abscess opened through the abdominal parietes, and all of them proved fatal; so that it seems doubtful whether such an opening be more favorable than one into the intestine or lung."

The author's remarks on the treatment of suppurative inflammation and abscess of the liver are, on the whole, judicious, yet, as we shall presently see, there are some things to be objected to. He advises that, if the inflammation be due to phlebitis, consequent on a wound, &c., means should be taken to prevent the ingress of more pus into the circulation. How is this to be effected? The strength of the patient is also to be supported, and the hepatic inflammation abated, as much as possible, by antiphlogistic means. The author then approaches the *quæstio vexata* of the value of mercury in hepatic affections:

"In this country, mercury has generally been resorted to, when the local symptoms have led to the suspicion that the liver was diseased; but, I fear, with no benefit to the patients. It has been well observed by Abercrombie, 'In the liver-diseases of this country, mercury is often used in an indiscriminate manner, and with very undefined notions as to certain specific influence, which it is supposed to exert over all the morbid conditions of this organ. If the liver be supposed to be in a state of torpor, mercury is given to excite it; if in a state of acute inflammation, mercury is given to moderate the inflammation and reduce its action." (p. 90.)

Now, greatly as we respect the authority of the late Dr. Abercrombie, and that of Dr. Budd, we must yet be permitted to express our opinion, that the use of mercury is defensible *both* in many states of torpor and in many also of acute inflammation of the liver. In many cases of tumid and tender liver, accompanied with equivocal marks of diminished secretion, as for example pale or white stools, no agent more rapidly reduces the volume and restores the action of the organ, than mercury. Again, he would be a bold man, who in almost any case of "acute inflammation" of the liver, except that caused by occlusion of the common duct, would *refrain* from using mercury, to a greater or less extent, and in conjunction, of course, with other means. We, for our parts, should consider that a practitioner who had failed to do so, had hardly given his patient all the professional aid which he might and ought to have done. We do not, of course, advocate the use of mercury *after* suppuration or abscess. Its very object is to prevent the occurrence of these, by resolution. After either of these events has taken place, mercury is to be withdrawn, if it have been used, and all our attention given to support the strength of the patient.

In reference to opening, by incision, abscesses of the liver, we *entirely* agree with the following observations of Dr. Budd; and the result of his experience of the method suggested by Dr. Graves, deserves publicity, though it would have been desirable had he entered into some further explanation and details.

"I would, then, never recommend opening an abscess of the liver, unless assured by circumscribed œdema, or a slight blush on the skin, that union had taken place between the integument and abscess. When these signs are wanting, and the skin has its natural appearance and colour, we can never be sure that adhesions have formed, and if we thrust a knife into the abscess, we run the risk of discharging the matter into the cavity of the peritoneum.

"Dr. Graves has ingeniously recommended a mode of proceeding, by which he supposes this danger to be obviated. It is, not to open the tumour at once, but to make an incision across the most prominent part of it through the abdominal muscles, so as to reach the peritoneum, without dividing it, and to fill up the wound with a pledgit of lint. The object of this is, to excite circumscribed

LIBRARY OF THE

Orleans Parish Medical Society inflammation of the peritoneum, which may produce adhesion between the reflected layer of the peritoneum and the layer covering the abscess. The abscess is then allowed to open of itself. I have tried this mode of proceeding twice, but with very unsatisfactory results." (pp. 92-3.)

The author, we need scarcely observe, condemns the use of the exploratory needle, as practised in India, and most truly observes:

"An occasional instance of success will, I fear, be a poor set-off against the cases in which the operation has done mischief, or failed of doing good." (p. 94.)

*Gangrenous inflammation.* The next section of this chapter is devoted to the consideration of "gangrenous inflammation" of the liver. It is very short, but we shall not make any remark upon it, except that Dr. Budd entertains the opinion that gangrenous inflammation is often disseminated from the extremities or from remote parts to the liver, in precisely the same way as suppurative inflammation is shown often to be in the former section, namely, by the septic matter entering a vein, passing to the liver, and there causing the same species of inflammation as the primary one. He gives a case (p. 99,) in which gangrene of the liver, lungs, and spleen, appeared to result from mortification of the toes from cold.

*Adhesive inflammation.* Section third (pp. 105 to 135 inclusive) is allotted to the description of adhesive inflammation of the liver. After briefly noticing or rather simply enumerating several causes of adhesive inflammation of the peritoneal covering of the organ, by which it is apt to be agglutinated to contiguous parts, the author proceeds (p. 107,) to consider "deep-seated adhesive inflammation." Sometimes the effused lymph is confined wholly to the cellular tissue surrounding the larger portal canals, the other parts of the viscus being healthy; at other times, the effusion of lymph takes place around the smaller twigs of the portal vein, those, namely, which divide the lobules. Hence the whole substance of the liver is rendered tough and somewhat white, by this deposition of coagulable matter, and the capsular as well as the peritoneal investment of the liver, are puckered by the new tissue contracting in the tracks of the *inter*-lobular veins, the lobules themselves retaining their volume. This form of adhesive inflammation and pathological change, called "hobnailed-liver" in this country, is designated "*cirrhosis*" by the French, from the Greek word *κιρρος*, fulvus, yellow, which is the colour which the liver presents, in this disease. This colour seems to be owing to the contractions caused by the effused lymph about the small gall-ducts interrupting the flow of bile among these, and thus giving to the lobules in which the bile is retained, a flavous hue.

*Cirrhosis.* In cirrhosis, the liver is at first much larger, afterwards much smaller than in health. In the first stage of the disease, the effusion of lymph and serum, together with the accumulation of bile in the portal capillaries, causes a large increase in the bulk of the organ; but when the inflammation has run its course, absorption removes the serum: those portions of the lobular substance, from which the escape of bile is permanently obstructed, by the contractions already referred to, become atrophied; and hence the liver subsides to a volume less than the normal. Dr. Budd mentions two facts illustrative of the great augmentation and subsequent diminution of the liver.

"Some time ago, in a case of advanced cirrhosis, I found a band of cellular tissue some inches in length, uniting the liver to the spleen. The adhesions must have formed when the organs were in contact, and have been drawn out as one or other contracted.

"In another case of advanced cirrhosis, I found the convex surface of the liver united to the diaphragm by tufts, or bands of false membrane, an inch in length. The parts of the liver at which these tufts were inserted, were *hollow or depressed*, and when all the tufts were divided, the surface of the liver was very uneven.

"Here, as in the case in which the liver and spleen were united, the adhesions must have formed when the surfaces were in contact, and the bands have been drawn out as the surfaces receded from each other. In both cases, these tufts or bands were evidence of the contraction of the liver, after adhesions had been formed. The degree of contraction being different in different parts, the surface of the liver becomes uneven." (114-15.)

Cirrhosis, both in this country and in France, seems to be chiefly caused by the constant use of alcoholic liquors. Andral considers that the hepatic disease is the consequence either of previously induced irritation on the duodenal mucous membrane, or that the alcohol is directly absorbed and carried into the liver by the portal veins. Dr. Budd regards the latter as the correct explanation. (p. 117.)

Cirrhosis is, commonly, insidious in its progress. It, like most hepatic diseases, is attended with structural change, is not accompanied with much or any suffering, unless the peritoneal investment of the liver is implicated. At other times, accidental circumstances may bring on a sudden access of inflammation, when, the train having been long prepared by spirit-drinking, the change constituting cirrhosis takes place promptly and with emphatic signs of inflammation. More usually, however, as we have remarked, the gradually increasing condensation of the liver and the abolition of the cells and their function in a great part of it, gives rise to sallowness of complexion, pale stools, and at length to ascitic symptoms, owing to embarrassment of the portal circulation. Ascites, indeed, Dr. Budd observes (pp. 121-2,) is an important symptom, because it occurs in few other diseases of the liver. "Frequently, however," he says, further, "together with the ascites, there is œdema of the legs, but unless there be some disease of the heart or of the kidneys, the œdema of the legs is always consecutive to the ascites." (p. 122.)

CHARITY HOSPITAL, MEDICAL LIBRARY.

A jaundiced colour of the countenance is, however, less common, and is less in degree, than in abscess of the liver, for in the latter disease, it is only a part or parts of the organ that are affected; the remainder being usually competent to their function: in the former disease, the whole liver is generally involved.

The livid complexion and the languor accompanying the disease are thus explained:

"The obstructed circulation through the liver serves also to explain in part, the sallow, dingy complexion, so constantly observed in advanced stages of cirrhosis. Part of the portal blood, instead of traversing the liver, finds another way, through the abdominal parietes, to the heart. This part of the blood cannot be purified, or freed from the constituents of bile, as it should be, and must consequently contaminate the whole mass of blood with which it is mixed. In this respect, cirrhosis offers an analogy to those cases in which there is a mixture of venous and arterial blood, in consequence of communication between the two sides of the heart.

"The emaciation and the loss of strength—other constant symptoms in cirrhosis

—depend perhaps in part on impairment of all the assimilating functions, by the habits of life that induce cirrhosis: but they are no doubt mainly owing to the obstructed circulation through the liver, and the imperfect secretion of bile.

"The obstructed circulation impedes, as we have seen, the absorption of water, and also of other substances that contribute to nutrition, by the veins of the stomach and intestines. Imperfect secretion of bile tends to impair nutrition in two ways. The bile, which no doubt performs an important part in digestion, flows in too small quantity into the duodenum, and digestion is in consequence imperfectly performed; and, on the other hand, some of the principles which should be eliminated as bile, remain in and contaminate the blood; causing languor and drowsiness, and weakening in some degree all the assimilating functions." (pp. 124-25-26.)

Cirrhosis is distinguished from chronic peritonitis, by the sallow hue of countenance, the amount of ascitic effusion and the permanency of it, and by the former occurring generally in spirit-drinking subjects, and usually not till after the 30th year. As regards *treatment*, it is obvious, that *after* effusion of lymph into the substance of the liver, no means are the least available. It is only therefore during the stage of the malady *prior* to effusion, in short, during the acute inflammatory stage, that treatment can be serviceable. But, it has been already remarked, that the disease is extremely obscure, in many cases, in its commencement. Hence Dr. Budd's remark is a very just one:

"In the person of a spirit-drinker, we should never neglect pain and tenderness in the region of the liver, especially if associated with some degree of fever." (p. 129.)

Bleeding of course will be requisite, but this measure ought to be practised with the recollection that drinkers are easily depressed by slight depletion. As Dr. Budd observes, an incautious bloodletting may, in such subjects, lead to delirium tremens or some even more formidable affection. Blistering, mercury judiciously employed, combined with the iodide of potassium, diuretics, light tonics, warm clothing, friction, a restricted use of alcoholic drinks, or a total abandonment of it, are the curative means which we must have recourse to. Friction, with mercury, and iodine over the region of the liver will also be expedient.

We pass over, entirely, section 4 of chapter ii, since great part of the information it contains has been incidentally given in the course of the quotations and observations already made. This section is occupied with the subject of "inflammation, suppurative and adhesive, of the branches of the portal vein, and with inflammation of the hepatic vein."

*Inflammation of the gall-bladder and ducts.* Section 5 (pp. 149 to 195 inclusive) is one of considerable practical interest and importance. In it are discussed various forms of inflammation of the gall-bladder and ducts. Catarrhal and suppurative inflammation is the species first handled. The former of these is a mild, and for that reason, a not easily detected disease. It is seldom or never fatal. "It happens, however, not unfrequently that on squeezing the hepatic ducts, a viscid whitish fluid oozes out, which, on examination, through the microscope, is found to be chiefly made up of the prismatic epithelial cells of the gall ducts." (p. 151.) The author then adds that the symptoms we might "*expect*" in catarrhal inflammation would be slight feverishness, and slight enlargement of the liver and jaundice, slight also, if the ducts were obstructed by the viscid secretion or the tumefaction of the viscus. In our opinion the disease and its symptoms and effects, are rather conjectural than ascertained.

Suppurative inflammation of the gall-ducts, is a severe affection. In a well-marked case, recorded by Dr. Olliffe of Paris, to which Dr. Budd refers, there were debility, slight nausea, daily rigors; but no jaundice. The gall-ducts through the entire organ were filled with pus; the gall-bladder was filled with pus and bile. Other organs seem to have been healthy.

In some cases part of the small gall-ducts become closed, in the course of suppurative inflammation, and the part beyond, that is, the lobular end of the duct, becomes dilated into a small pouch, being "converted into small permanent cysts, filled with a glairy fluid, more or less tinged with bile." (p. 154.) These may be mistaken for tubercles.

However, the cystic and common ducts and the gall-bladder are more frequently the seat of inflammation than the hepatic ducts. At p. 155, Dr. Budd quotes a case from Andral, which he represents as "the best example he has met with of acute inflammation of the common duct only." From this language, we are led to infer that the common duct was the primary seat of inflammation, and indeed the only diseased part. But, in our opinion, Dr. Budd has entirely mistaken the nature of the case. Andral's words are: "the coats of the common duct were much thickened and easily torn, and the canal almost closed;" and elsewhere, "the entrance of the common duct was marked by a small round tumour, rising three lines above the surface of the intestine, and pierced at its summit by a capillary orifice, the opening of the duct." (p. 157.) Not a word about *inflammation* of the common duct. But—"*the inner surface of the duodenum was intensely red,*" (same page)—and here, according to our belief, was the *punctum saliens* of disease. It confirms this view, that the disease commenced after indulgence at table. The tumefaction of the duodenum had closed the common ducts, but there is no proof that the duct was inflamed, and the current of bile, obstructed, had ruptured the hepatic duct, just beyond its junction with the cystic, giving rise to fatal peritonitis in consequence of the escape of the bile into the abdominal cavity.

In this case of Andral's, and in another quoted by Dr. Budd from the same authority, there was a moveable pear-shaped tumour, immediately under the false ribs. This was the distended gall-bladder. Dr. Budd's comments on these two cases are deserving of quotation:

"There can be little doubt that this case, like the former, was one of acute inflammation of the common duct, not extending to the gall-bladder. The symptoms in these cases were just what might have been expected: pain in the situation of the duct, followed, at the end of one or two days, by jaundice and by distension of the gall-bladder; a certain degree of fever; constipation. It is worthy of remark that in neither case does Andral notice among the symptoms, vomiting, or nausea, or rigors

"It is probable that similar cases now and then occur, and are treated as inflammatory jaundice, without their real nature being discovered. The symptoms differ from those of ordinary cases of jaundice, chiefly in the pain being limited to a small spot in the situation of the common duct, and in *the early appearance of a large, moveable, pear-shaped tumour,* not painful or tender, which may be recognized by these characteristics as the gall-bladder distended from closure of the common duct. The absence of pain or tenderness of the tumour, shows that the gall-bladder is not inflamed.

"If the inflammation should involve the cystic and hepatic ducts, as well as the common duct, distension of the gall-bladder would perhaps not take place, and the symptoms would be merely those of inflammatory jaundice." (p 158.)

Inflammation of the gall-bladder, and even permanent structural change in it, are not necessarily fatal, provided the cystic duct remains patent.

Rokitansky has observed, in "the secondary fever of cholera" and as a result of typhoid fever, what he calls *croupal* or plastic inflammation of the mucous membrane of the gall-bladder and ducts. "It produces," to use Dr. Budd's words, (p. 163,) "within the gall-ducts, membranous tubes, in which the bile (?) forms tree-like concretions." It is a rare affection.

The author next makes a few remarks on, and gives a case from his own practice, and that of Andral, and Cruveilhier, and Mr. Bowman respectively, illustrative of ulceration of the gall-bladder. This form of disease and gall-stones are often found coexistent; and though, no doubt, the latter sometimes cause the former, yet Dr. Budd well remarks, that in many cases "it seems most reasonable to refer both ulcers and gall-stones . . . . to an unhealthy state of the bile." (p. 164.) This inference is legitimate, in those cases in which the gall-stones are found not resting on the ulcers, and when it is recollected that the gall-stones *float* in the bile, and are found thus floating after death.

It is important to keep in mind, "that ulceration of the gall-bladder, like ulceration of other mucous surfaces which return their blood to the portal vein, may lead to scattered abscesses of the liver." (p. 175.)

At pages 180 et seq. we have some observations on occlusion of the cystic and common ducts. Closure of the cystic ducts most commonly occurs from a biliary calculus, which, floating in the bile of the gall-bladder, is carried into the duct, gets impacted there: inflammation may then set in and fix it, or it may pass through the walls of the duct. If the stone remain fixed in the duct, or should the duct be permanently closed by any other cause, the bile in the gall-bladder is absorbed, (supposing the bladder to have been healthy,) and a glairy fluid alone remains. The bladder also shrinks to the size of the smallest pear, or even to that of an almond. But, from various cases, it appears that the effect of closure of the cystic duct simply, may be but little detrimental to digestion, to health, or to longevity.

Occlusion of the common duct is an occurrence much more serious and urgent. If the duct have been closed suddenly, and the secretion of the bile have been going on actively, and in a liver comparatively healthy, the rapid distension of the gall-bladder and ducts may be such, that rupture of one or other ensues. Not a few cases of this kind are on record. If the closure of the common duct has been gradual, the bile, of course, slowly distends the gall-bladder and ducts throughout the liver, which thereby acquires a deep brown colour. It may happen (in this slow form of occlusion) that before rupture anywhere takes place, the structure of the liver is destroyed, and the nucleated cells are either broken down or absorbed. This occurred in a case recorded by Dr. Thomas Williams, and still more remarkably in a case recorded by the author himself. (pp. 183-7.)

In cases of complete closure of the common duct the liver generally enlarges at first, but by and by, if life lasts for some time, it subsides into a size more or less below the natural.

Though our limits will not permit us to quote the case referred to in the conclusion of the second last paragraph, we must yet give the reader the benefit of one or two useful practical comments which the author makes on it:

"The constipation, and the relief she derived from purgatives, and once from diarrhœa, that occurred without purgative medicine. Much of the pain and tenderness of the belly of which she complained, was probably owing to distension of the intestine by fæces and gas, and to irritation of its mucous membrane by the contact of matters chemically different from those natural to it.

"The fetid urine, which was at times turbid with pale lithates, but never had a *pinkish sediment*. The absence of a pink sediment may help to distinguish such cases from cases in which the common duct is closed by the pressure of a cancerous tumour, and in which a sediment of this tint is often observed in the urine.

"But perhaps the most striking circumstance of all was that, although for a long time before death the liver must have ceased to separate bile from the blood, there were no symptoms of cerebral poisoning, and the mind remained clear to the last. This circumstance will appear still more remarkable, if we compare this case with other cases in which suppression of bile is attended with delirium, or with stupor and convulsions, soon ending in fatal coma. Dr. Alison, in a paper published in the Edinburgh Medical and Surgical Journal for 1835, has collected many cases of this latter kind, and from a review of them he concludes that it is jaundice from *suppressed secretion*, and not from obstructed gall-ducts, that is peculiarly, if not exclusively, liable to be followed by delirium, coma, and speedy death. He explains this, by supposing that 'the retention in the blood of matter destined to excretion, is much more generally hurtful to the living body than the *reabsorption* into the blood of matters which have been excreted at their appropriate organs, but not thrown out of the body, in consequence of obstruction at their outlets.' The fact is, I believe, correct, but Dr. Alison's explanation is not satisfactory, since in this case, for a long time before death, there could have been no bile secreted, and yet there was no disorder of the brain.

"The case further shows us, that the secretion of bile by the liver is not immediately necessary to life; that a person may live a considerable time when the liver, as an instrument of secretion, is completely destroyed. This destruction of the secreting element of the liver proves fatal, however, in the end, by impairing nutrition, and causing gradual but progressive wasting." (pp. 188-90.)

Dr. Budd refers to a case of Mr. Busk's, on board the Dreadnought, in which, in the author's opinion, no bile had passed into the bowels during four years! (p. 190.)

Dr. Budd repeatedly, throughout his work, refers to the fact that disease of the gall-bladder, more especially fatty degeneration, is "always attended with a large secretion of cholesterine, . . . . which frequently leads to the formation of gall-stones, and all the evils they occasion." (p. 191.) The author sums up his history of the inflammation of the gall-bladder and ducts, with the remark that the immediate cause of most of the forms of inflammation of these parts, is the passage of irritating bile or gall-stones.

The author's remarks on treatment are concise and rather vague. Leeches, blisters, mercury, not with the view of obtaining its "more powerful and constitutional action," (p. 194,) but of increasing the quantity of bile, and rendering it less irritating, which the author conceives mercury, judiciously administered, effects: diluents which, "by filling the stomach" may "empty the gall-bladder by their pressure," and also by "passing out again of the circulation by the liver," may thus "dilute the bile;" soda which, in the catarrhal form of inflammation of the ducts, may "probably render the secretion from them less viscid;" taraxacum which, according to the author, "like cholagogue medicines generally, is more likely to be under than over-rated," (p. 195)—(is such Dr. B.'s opinion?)—these are the means suggested in the work before us for the cure of the lesions to which our attention has been directed.

Chapter third, which occurs next, is one of considerable interest.

*Fatal jaundice.* It occasionally happens that fatal jaundice occurs in cases where, on a post-mortem examination, the gall-ducts are found patent, nor are there any of those effusions which indicate that inflammation has been at work. In such cases we must infer that the jaundice is not caused by obstruction to the passage of bile, in so far as the hepatic ducts throughout are concerned; nor can we suppose, of course, that the morbid changes which we sometimes detect in the liver, are owing to the current of bile being thrown back. On the contrary, the jaundice, in the cases we are now discoursing of, is the consequence of no bile being secreted, the cell-laboratories in which that secretion is carried on being either greatly diminished in number, or in great part destroyed. In some cases, however, while the cell-apparatus remains, so far as we can judge, in perfect condition, yet these secrete no bile. This is a mysterious paralysis or stoppage of the *function* of the cells, the precise cause and nature of which our present pathological knowledge does not enable us to appreciate.

In those cases in which the cells are diminished in number or disorganized, there are sometimes seen, on examining sections of the liver with the microscope, irregular collections of oil-globules, biliary particles, and amorphous granules.

The functional suspension of secretion is often due to sudden, violent, and depressing moral emotions; but according to the author, "it is far more frequently (?) produced by some poison introduced from without or generated in the body by faulty assimilation or digestion." (p. 224.) The author notices one point in diagnosis which we shall quote:

> "When delirium, or coma, or convulsions supervene, we may be almost sure that the jaundice results from suppressed secretion; because these symptoms seldom occur in jaundice that results from mere obstruction of the ducts." (p. 255.)

In regard to this form of hepatic disease, we cannot find fault with the author's customary brevity of therapeutical remark:

> "Until more is known of the causes of this form of disease, and until it can be detected with more certainty, we cannot expect to have satisfactory proofs of the good or ill effects of particular plans of treatment. The conclusion that may be most safely drawn from the foregoing cases is that, in some instances, coma may probably be prevented or removed, and the life of the patient saved, by active purging." (pp. 225-6.)

We pass over entirely sections 2d and 3d of this chapter (third,) in which are respectively discussed fatty degeneration and scrofulous enlargement of the liver. In section 4th we come to the extremely interesting subjects, excessive and defective secretion of bile and unhealthy states of that secretion.

*Biliary derangement.* The causes of derangement of the biliary secretion are stated to be structural disease of the liver, or morbid states of the portal blood from medicines, unwholesome food, "faulty digestion or assimilation," or the deficient action of some other excreting organ. (p. 256.)

*Excessive secretion of bile* is characterized by bilious stools, ardor fæcum, nausea occasionally, bitter taste, yellow tongue. A more severe form of the disease conjoins to the symptoms just enumerated, fever more or less lively. The fever is probably owing to the acrimony of the bile irritating in its passage the gall-ducts and small intestines; for, observes the author (p. 258,) "There can be little doubt that the bile, while it is increased in quantity, is also altered in quality and irritating," a conclusion to which we only partially assent. The cure for these forms of biliary irritation and excitement are saline purgatives, *no* calomel (as we think,) though the author and Mr. Annesley recommend it; diluent drinks, and phlebotomy or cupping, which Mr. Annesley directs, but which we think need be resorted to *only* if there is *considerable* fever and hypochondriac pain, provided purgatives and abstinence be practised.

To the medicinal treatment the author appends certain hygienic suggestions so proper and useful, that we shall present them in his own words:

"These measures are generally sufficient for the time, but they do not strike at the root of the evil. Exemption from future attacks, and from the manifold and greater evils to which these disorders may lead as age advances, can only be procured by a change of habits. One of our objects in directing this should be to increase the amount of oxygen inspired, and thus to consume in respiration, or burn off, materials that would otherwise be left for the liver to excrete. The means most efficacious for this purpose are sea-voyages, riding, or other exercise in the open air, well-ventilated rooms, early rising, the cold or shower-bath, &c. Too much indulgence in sleep, which so much reduces the activity of both respiration and circulation, must be especially injurious, more particularly in rooms that are ill-ventilated, as most bed-rooms are.

"Another object of equal or still greater importance, should be to limit in the food the supply of those materials—such as spirituous liquors, butter, cream, fat, sugar—which contribute directly to form bile, or which increase the quantity of bile indirectly, by serving as fuel for respiration. Some of those aliments, as cream and porter, for instance, seem to be not only pernicious in this way, but also by directly embarrassing the secreting function of the liver.

"From these considerations it follows, that it must be especially injurious for persons who suffer from the disorders we are considering, to indulge in sleep immediately after a full meal. To lessen by sleep the activity of respiration at the very time when the materials consumed in this process are being poured in large quantity into the blood, must lead in a twofold way to accumulation of bile in the system, and favour the occurrence of a bilious attack. In this way may be explained the ill effects of suppers in disorders of this class, and the well-known fact that a single indulgence of this kind may bring on a bilious attack, in a person predisposed to it." (p. 259.)

*Deficiency of bile* is the next derangement. This, according to the author, may be of two kinds: one in which enough of bile is eliminated by the liver to purify the blood, but not to effect digestion properly; another, in which there is not enough of bile secreted either to purify the blood or produce perfect digestion. In the former case, digestion is slow, nutrition imperfect, the bowels irregular, "and the contents of the large intestine often become too acid." (p. 260.) Dr. Budd attributes the disease to too great abstemiousness, (hear this, ye abettors of feeble, insipid diet!) and regards the cure to lie in "less abstemiousness." Alas! how few diseases can boast of the *cause!* to how few is the *cure* suitable!

Another form or variety of this first kind sometimes occurs in young people. There is diarrhœa which resists all sedatives and astringents. The discharges are entirely *un*tinged with bile. As soon as the bile flows, the disease stops. Dr. Budd thinks the disease owing to irritating matter causing a temporary spasm or inflammation of the mouth of the common duct. The irritating matters referred to the author states to be morbidly acid, and that relief is afforded by magnesia. The author further quotes from Dr. Prout a description of a somewhat similar spasm of the ducts, attended with intolerable headache, which ceases so soon as the released bile is supposed to have reached the cæcum, showing the affection probably to depend upon unnatural acidity of the contents of that portion of the intestine.

The second form of deficient secretion of bile is when this fluid "may be in excess as regards the intestines (p. 263,) .... and yet may be in too small quantity to purify the blood, and the complexion be bilious or sallow." We have noticed frequently such a state of matters. The author states the cure to be "a dose of calomel and a few brisk purgatives." We have not found the affection always yield to such homely treatment.

The second and more serious variety of this second form of deficient secretion of bile is, when the inadequate action of the liver results from any of the inflammatory changes of the organ, noticed in the former part of this article, such as adhesive inflammation of the areolar tissue surrounding the branches of the portal vein, and impeding the passage of bile along them, or interstitial fibrinous deposit, as in cirrhosis; or else from any cause by which the function or structure of the nucleated cells is abolished. In such cases, of course, the defective secretion of bile is a mere effect of a specific change, and can only be cured by the removal of that change.

But though, as the author observes (p. 264 et seq.) health cannot, in these circumstances, be perfectly restored, something may be done to prolong life and to palliate the situation of the patient. All articles of diet tending to favour the formation of bile are to be abstained from; much pure air is to be inhaled, by which the lungs are made to supplement the liver, thus relieving the latter organ of some labour; a pill of aloes and rhubarb to be taken habitually at dinner; and certain medicines to be employed, some of which appear to act by making less secretion of bile necessary; others by increasing the activity of the liver, thereby making up for the disorganized condition in which part of the organ is, by the augmented action of those portions of it which are still sound. Of the former sort of medicines nitro-muriatic acid, used externally and internally, is a specimen: among the latter, the author cites mercury and taraxacum.

A short notice of "unhealthy states of the bile" next follows. On this subject there seems to be extremely scanty information. In cirrhosis and the fatty degeneration of the liver, the colouring matter and the bitter taste of the bile are somewhat wanting. But the author has found the same peculiarities in cases in which the liver itself was quite healthy, but where there was granular kidney in one instance, and purulent phlebitis with pulmonary abscesses in another. (pp. 267-8.)

In other cases the bile is dark and inspissated. This is more especially the case in diseases of India, where the bile seems to be secreted in a more concentrated form, if the word may be used, than with us, and where the occasional irruption of such bile, after temporary retention in the gall-bladder and ducts, often causes great and even dangerous disturbance.

*Gall-stones.* Section 5 is devoted to the consideration of gall-stones. Our notice of this section and of the remainder of the work must be exceedingly brief.

There are two kinds of gall-stones, the encysted, those namely, found in the substance, as it seems of the liver, and those found in the gall-bladder. The former, called hepatic gall-stones, are usually very small, and merely consist of inspissated bile and mucus; they result from inflammation and stoppage of some hepatic duct or ducts on the side next the gall-bladder. Gall-stones also of similar kind, namely, of inspissated biliary matter mixed with mucus, are found in the gall-bladder itself. When in this situation, and if unincased in cholesterine, they show that the bladder is healthy, for otherwise cholesterine would be deposited around them. When the gall-stone, as sometimes happens, is entirely composed of cholesterine, it is white, and resembles a ball of camphor. (p. 274.)

Gall-stones are sometimes single, and the size of a hen's egg; sometimes in numbers of several thousands and proportionally small. Where there are but a few, they come to resemble (as remarked by Haller,) the small bones of the wrist, in consequence of the attrition one with the other.

It is an important fact that the gall-stones found in the same bladder usually resemble each other. Their nuclei and laminæ are the same: if one shows a layer of cholesterine, all do so. Hence we must infer they were all formed simultaneously. To what serious and permanent consequences may even a temporary abnormal condition of the bile give rise!

Gall-stones always indicate a deranged condition of the bile at the time they were formed. They almost always consist of cholesterine, and biliary matter and mucus. They seem seldom to result from organic disease; never, according to the experience of Dr. Prout and the author, from the granular change of the organ caused by dram-drinking. They are sometimes, however, found associated with cancer of the liver, or with the cancerous *diathesis.* The deposition of cholesterine seems to be associated with fatty degeneration, not of the liver, but of the coats of the gall-bladder.

There seems a greater liability in women than in men to gall-stones. They most usually occur in persons of full habit, who use rich food and take little exercise. Dr. Prout remarks a relation between the tendency to cholesterine gall-stones and the uric-acid deposits.

A gall-stone may remain in the bladder or even in the ducts (provided it does not interrupt the passage of bile,) for an indefinite period without causing any pain, or a single symptom to indicate its existence. Its passage, however, along the cystic and common ducts, is usually attended with exquisite suffering. The pain comes on suddenly, and is usually not continued but paroxysmal. There is often vomiting. If the stone is long in passing, and becomes impacted in the common duct, jaundice ensues. If it remains permanently impacted there, death may take place from coma, or inflammation may occur in the duct, the stone along with bile escaping into the cavity of the abdomen; or, what more usually happens, adhesion takes place with some neighbouring organ, often the stomach or duodenum, into one or other of which the calculus eats its way. Another occasional result is that the gall-bladder, suddenly distended beyond what it can bear, bursts, and its contents escaping into the abdominal cavity, the patient perishes from acute peritonitis.

The most safe and, fortunately, the most common occurrence, is that the stone slowly and painfully passes the *straits* of the common duct, and emerges into the duodenum, with *instantaneous* and *complete* cessation of the patient's sufferings.

Of course inflammation and fever may occur in the course of this process, if protracted and severe. To combat these is almost all we have to do or can do. Dr. Prout suggests, as a mode of giving relief, large draughts, repeated, of hot water (if necessary with some laudanum in it,) containing carbonate of soda. Fomentations with hot water and poppies, or, as some direct, pounded ice, is to be applied to the epigastrium. A warm bath also gives relief sometimes. Leeching of the hypochondriac region is proper, if the pain is extreme. Antispasmodics are also proper with a view of promoting dilatation of the duct.

In regard to stones presumed to remain still in the bladder, and in respect to the formation of new ones, Dr. Budd thinks that *soda* may have a tendency to accomplish the solution of the former, by forming "a soluble compound of cholesterine." (p. 296.) The second end is to be attained by regulating the bowels by "the habitual use" of rhubarb or rhubarb and aloes, by mild saline purgatives, as Püllna water; by keeping up the action of the skin by the warm bath; lastly, by an occasional use of mercury; all which suggestions have in general our concurrence.

Chapter iv is occupied with diseases of the liver which result from some growth foreign to the natural structure; and section first is devoted to cancerous tumours and "encysted, knotted tubera" of the organ. (p. 299.)

*Cancer of the liver.* We do not mean to pause upon these matters. One remark of Dr. Budd's, however, takes us by surprise: "No serious organic disease of the liver is in this country (at least among people who have never drunk hard,) so frequent as cancer." Can Dr. Budd furnish any statistical proofs of this?

Cancer appears sometimes ubiquitously and simultaneously; sometimes it is plainly successive and disseminative by the lymphatics. Dr. Budd shows this in various parts of his work. (p. 310, et seq.) We shall quote some brief remarks on the diagnosis:

"We are ignorant of the conditions which dispose to primary cancer of the liver, or which immediately excite it, so that in the diagnosis of this disease, we are little helped by knowing the previous habits of the patient, or the circumstances in which he has lately been placed. We know only that the disease does not occur before the age of thirty-five. In persons above this age it can only be discovered by the intrinsic import of the symptoms. But in the early stages of the disease, and while the liver is still shielded by the ribs, the symptoms are vague, and such only as are common to various derangements of this organ. They may justly excite our fears, but they cannot give us assurance that the liver is the seat of cancer.

"The most significant symptom is enlargement of the liver. When this comes on in the middle period of life, and especially when it is progressive, and when other conditions that may equally give rise to it are wanting,—when there is no obstacle to the circulation in the chest, when the patient is not consumptive, and when his habits have not been such as to lead us to suspect that he may have cirrhosis,—it affords of itself strong presumption of the presence of cancerous tumours. When the liver is of very great size, and its surface can be felt to be nodulous or uneven, there is no longer room for doubt.

"Another symptom which is of very frequent occurrence, and which may help us to distinguish this disease from some others in which the liver is likewise enlarged, is constant pain and tenderness.

"A small, permanent collection of fluid in the cavity of the peritoneum, when there is no reason to believe it to be the result of cirrhosis, is another significant token of the presence of cancerous tumours in the liver. A large quantity of fluid in the peritoneum is less significant of itself, and it may even increase the difficulty of diagnosis, by preventing our feeling the large and nodulous liver.

"When cancer of the liver is consequent on cancer of some other part, its detection is much easier, because, from our knowledge of the frequent dissemination of cancer, symptoms which are in other circumstances trivial, then acquire great significance. In a woman who has ulcerated cancer of the breast, with the general symptoms of the cancerous cachexy; or in one who has cancer in the uterus which has eaten into the intestine; or in a person who has presented for some time the symptoms of cancer of the stomach,—pain and tenderness in the region of the liver, or a slight increase in its volume, with jaundice, or slight ascites, or even one of these symptoms, are evidence enough that cancerous tumours have formed in this organ. The same symptoms, occurring soon after an injury to the head, or after amputation of the leg or arm, together with the constitutional symptoms of suppurative phlebitis, would scarcely leave a doubt that abscesses were forming in the liver. Our conclusions are drawn, not so much from the intrinsic value of the symptoms, as from the significance which these derive from the circumstances under which they occur." (pp. 325-7.)

*Jaundice.* Though jaundice is justly stated by the author to be a mere symptom, and though it is, on various occasions, incidentally referred to in previous parts of the work, yet Dr. Budd thinks it proper (and justly so,) to devote to its consideration the concluding chapter of his work. He here collects into one view all the notices on the disease scattered through other parts of his volume. We shall endeavour to give an extremely condensed recapitulation of a few of the principal particulars.

Jaundice, as is well known, arises either from the retention of the colouring matter of the bile in the blood, or from its absorption. In many of the circumstances causing jaundice, the yellow hue of the skin has both of these origins. It seems still somewhat doubtful whether bile, as bile, exists in the blood, or only its elements. Lecanu, whose researches into the composition of the blood have been of a very rigorous kind, has only detected the colouring matter. The non-secretion or the absorption of bile is, after a time, attended with diminution of the number of the red globules; but this effect seems to be rather an indirect consequence of impaired nutrition, than owing to any immediate influence of the bile on the blood.

In approaching jaundice, the presence of bile in the urine is sometimes detectible, before the yellow hue of skin is manifested, by the test of sulphuric acid, which causes a greenish colour in the urine, changing to purple or red.

As the yellow colour of the skin is some time of showing itself, so it often remains after traces of bile have disappeared from the urine and *re*appeared in the fæces; in short, after the action of the liver has been restored. We must therefore be cautious not unnecessarily to persist in the use of mercury or other measures, on account of the continuance of the yellowness of skin merely, which diaphoretics and warm baths will gradually remove.

In regard to the causes of jaundice Dr. Budd, after mentioning gall-stones as one of these, observes: "A more frequent cause of jaundice from obstructed gall-ducts is cancerous disease of the liver, or of the pancreas," which he adds is permanent. (p. 381.) We can scarcely admit with Dr. Budd as to the greater frequency of jaundice from cancerous disease than from gall-stones.

Many cases of jaundice are owing to the structural affections of the liver described in the earlier part of Dr. Budd's volume. These it is needless to recapitulate. The general inference deduced by the author is of practical importance, namely, that in jaundice, springing as it may do from such a great variety of causes, and these of such various shades or rather kinds of disease, the prognosis must be very difficult until we have arrived at certainty as to the lesion on which the jaundice depends. This is in some cases much more easy than in others. The previous habits of the patient in a case of cirrhosis; the sudden incidence of symptoms marking the passage of a gall-stone; or the gradual appearance and persistent presence of jaundice without pain or stupor in the course of the disease, indicating some gradual obstruction of the ducts, and not a case of suppression, that is non-secretion of bile, which is always attended with coma; the accompanying circumstances now detailed, may enable us, in cases characterized by them, to come to pretty definite conclusions as to the cause of the jaundice. But in many other cases the diagnosis and prognosis will be far from easy.

Jaundice, *per se*, requires of course no remarks as far as regards treatment, which must have in view the cause, and not the symptom.

We have thus brought to a conclusion our notice of Dr. Budd's able and practical volume. We have no hesitation in pronouncing it an opportune and useful publication. Were we to find any fault, we should say that, in the treatment of the subject, pathology commands too much attention, therapeutics too little. The work is well written, at least perspicuously written; it pretends to no peculiar elegance, liveliness, or finish of style, nor to any great philosophy of thought or doctrine. But the language is clear; there are no redundant matters or language; and on the whole, this treatise on "Diseases of the Liver" is one which none but a man well versed in his profession could have composed. And we do not doubt that the tolerably full digest which we have given, and the quotations we have made, will induce our readers to seek in the work itself that large amount of valuable pathological facts and inductions which we confidently promise them.

---

